# Dendritic cells loaded with tumor derived exosomes for cancer immunotherapy

**DOI:** 10.18632/oncotarget.20812

**Published:** 2017-09-11

**Authors:** Hongyu Liu, Ling Chen, Yaojun Peng, Songyan Yu, Jialin Liu, Liangliang Wu, Lijun Zhang, Qiyan Wu, Xin Chang, Xinguang Yu, Tianyi Liu

**Affiliations:** ^1^ Department of Neurosurgery, Chinese PLA General Hospital, Beijing 100853, China; ^2^ Key Laboratory of Cancer Center, Chinese PLA General Hospital, Beijing 100853, China; ^3^ Department of Endocrinology, Chinese PLA General Hospital, Beijing 100853, China; ^4^ Department of Clinical Laboratory, Weihai Municipal Hospital, Weihai 264200, Shandong, China

**Keywords:** tumor derived exosomes, dendritic cells, immunotherapy

## Abstract

Exosomes are vesicles that can be secreted by many types of cell and released into the extracellular space. Studies have found that tumor derived exosomes (TEXs) can promote tumor growth and metastasis, as well as inhibit immune response through transferring their genetic information to the recipient cells. Given their functions in tumor progression, TEXs are considered as promising biomarkers for early detection of human malignancy. Dendritic cells (DCs), a type of antigen presenting cells, can induce tumor-specific T cell immune responses in carcinogenesis. Growing evidences have demonstrated that the matured DCs induced by TEXs exhibit enhanced anti-tumor effects that may be applied for cancer immunotherapy. Thus in this review, according to the previous studies, we summarized the effects of DCs loaded with TEXs in cancer immunotherapy.

## INTRODUCTION

A small vesicle was firstly observed in sheep reticulocytes in 1983 [1]. Later Rose M. Johnstone defined it as exosomes [2]. Exosomes are 30–100 nm vesicles, which are secreted by almost all types of cell including tumor cells [3, 4]. They are composed of proteins and nucleic acids that are associated with donor cells [5]. Exosomes also have specific membrane markers such as tetraspanins (e.g. CD9, CD63, CD81), heat-shock protein (Hsp70), major histocompatibility complex molecules (MHC I and II) and costimulatory molecules [6]. In 1996, Raposo et al. firstly reported that B-cell-derived exosomes induced antigen specific MHC class II restricted T-cell responses [7], and then Zitvogel et al. demonstrated that dendritic cells (DCs) pulsed with tumor-derived peptides elicited potent antitumor T-cell response in tumor-bearing mice in 1998 [8]. These results have indicated that exosomes have potential capacities for cancer immunotherapy. In 2001, Wolfers et al. found that tumor-derived exosomes could be used as a source of shared tumor rejection antigens for CTL cross-priming [8]. Based on the findings, tumorous immunotherapies researches were carried out in various malignancies, such as melanoma, glioma, hepatocellular carcinoma and renal cell carcinoma [9–14]. In this review, we will summarize the function and application values of DCs loaded with tumor derived exosomes in cancer immunotherapy, as well as the related mechanisms.

### Exosomes in cancers

**Figure 1:**
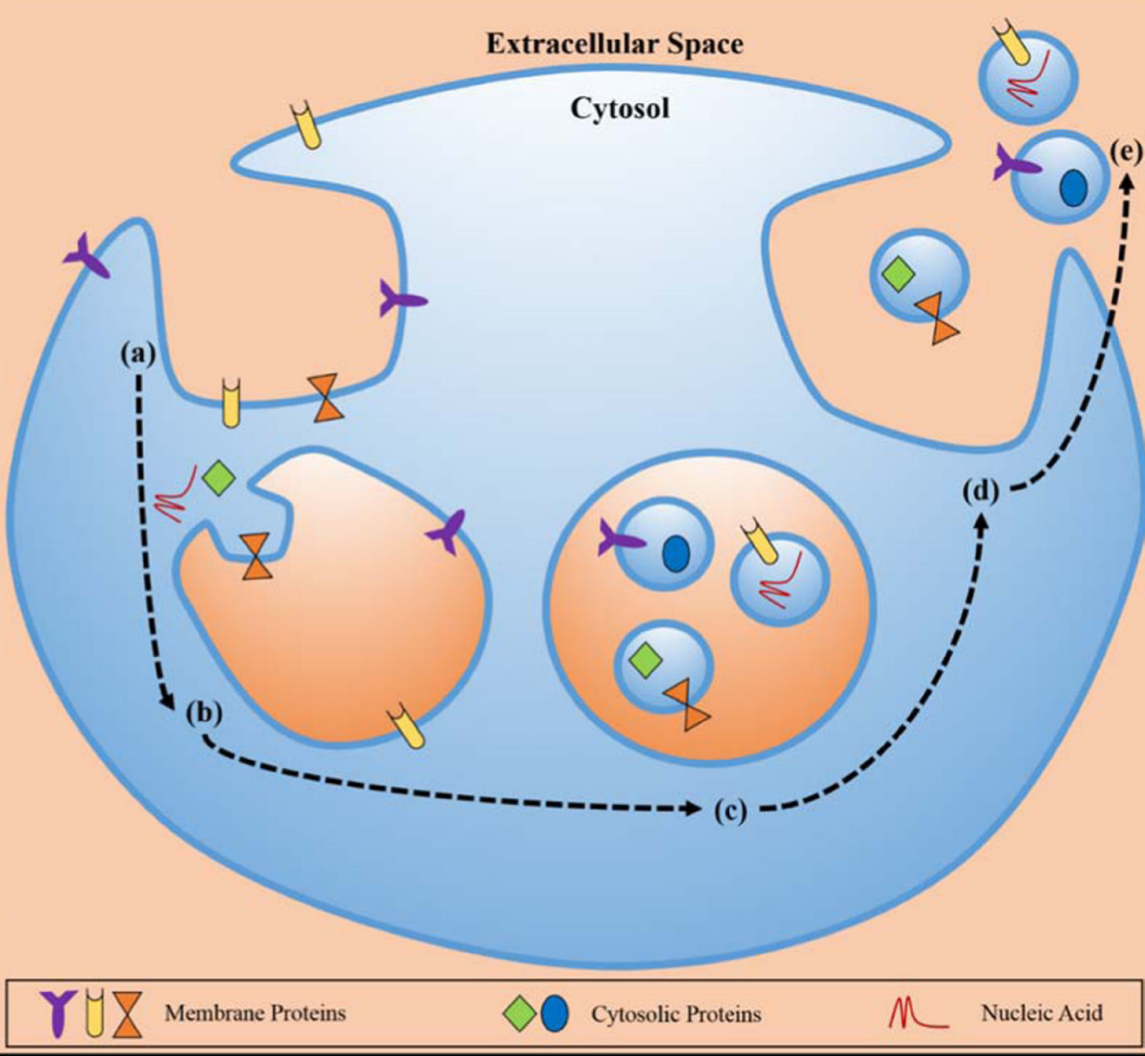
The biosynthetic pathway of exosomes Exosomes was composed with proteins, cytosolic proteins, and necleic acid. Exosomes formation occurred at the membrane via enodocytosis, and related to the cellular environment through exocytosis (The figure from Munson et al. [73]).

**Figure 2:**
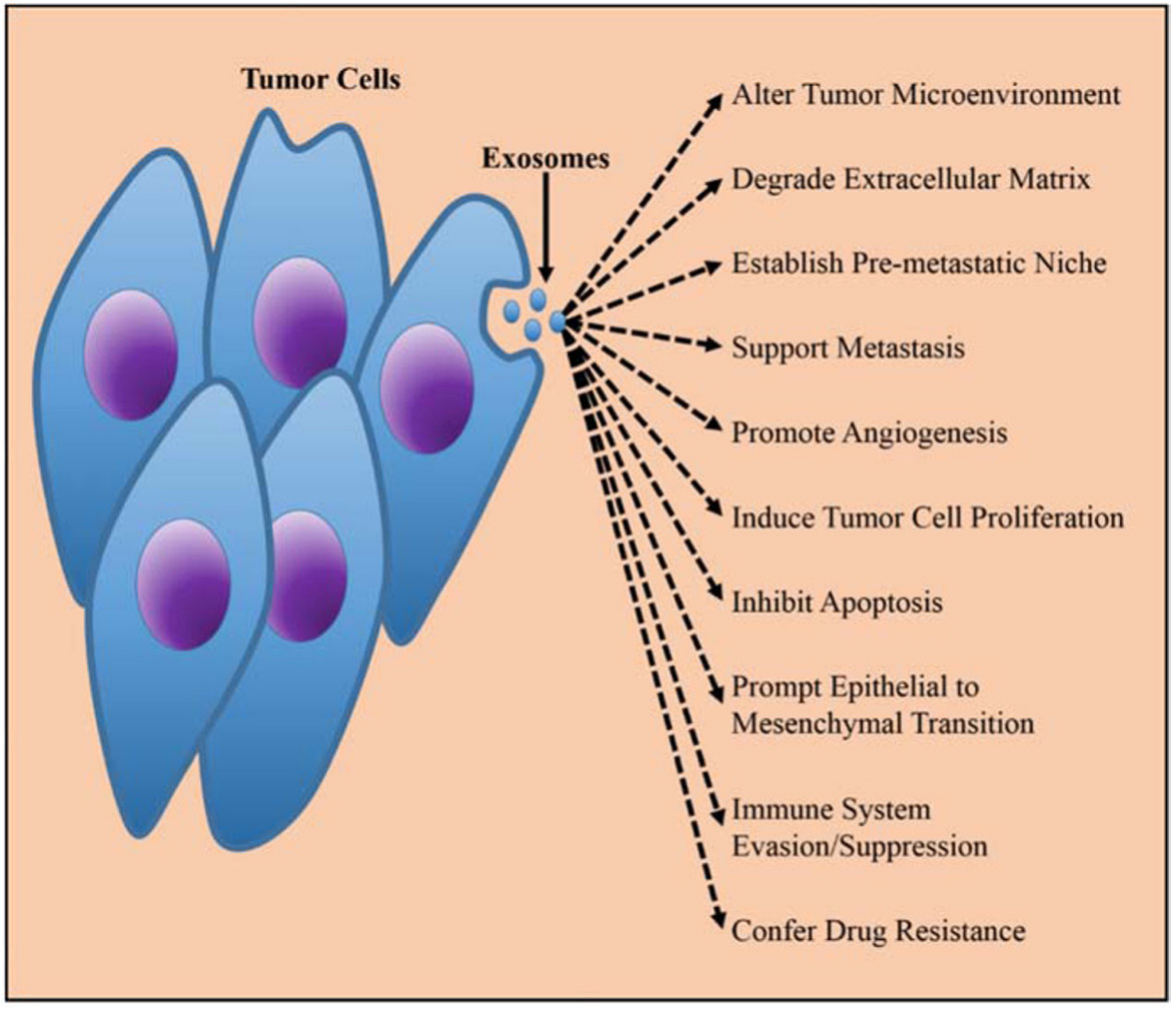
The biological function of TEXs in tumorigenesis Released by the tumor cells, TEXs played important promoting roles in malignant progression of tumors via improving tumor environment, supporting metastasis, immunesuppressive, etc. The figure was from Munson et al. [73].

Exosomes are formed through endocytosis in cell membrane, and then resulting in formation of multivesicular bodies (MVBs) that can fuse with plasma membrane and be released to the extracellular space or body fluids via exocytosis (Figure 1). Exosomes can carry the contents of their host cells, including miRNAs, proteins, lipids, even DNAs. Therefore, exosomes maintain the part function of their host cells. Released from malignant cells, tumor derived exosomes (TEXs) can promote tumor growth through inhibiting differentiation of bone marrow cells [15–17], changing physiology of macrophages [11, 18, 19], reducing NK cell cytotoxicity [20–22], and regulating the role of T cells [23–29]. TEXs also plays an important role in tumor metastasis through transporting their contents to recipient cells thus affecting intracellular signal pathway [30–35] (Figure 2). Compared with exosomes released from normal cells, the quantity of TEXs is significantly more because the malignant cells secrete about 10 times more exosomes than the normal cells (Table 1). Abundant of TEXs in tumor microenvironment may enhance tumor immune invasion via inhibiting the function of effector T cells and NK cells. TEXs may also play inhibitory roles in differentiation of DCs. TEXs can help the tumor cells escape from host immunosurveillance, thus facilitating tumor growth and metastasis [17, 20–22].

**Table 1:** The comparison between TEX and normal cell derived exosomes

	TEXs	Normal cell derived exosomes
Origin	Tumor cells	Normal cells
Biological components	mRNAs, miRNAs, lnRNAs, proteins, antigens, DNA, etc	mRNAs, miRNAs, lnRNAs, proteins, antigens, DNA, etc
Quantity	About 10 times more than the normal cell derived exosomes	less than the numbers of the TEXs
Characteristic protein	Cancer-specific antigen	the proteins or protein family representing their endosomal origin: for example exosomes from DCs carried abundant of MHC class II and CD86
Function	immune inhibition; promoting angiogenesis, growth, invasion, metastasis; drug resistance	intercellular communication; anti-tumor action; immune-stimulatory effects;
Application	tumor biomarkers and targeted therapy	targeted delivery; cancer treatment

In the early time, researchers focused on their potential as diagnostic markers because TEXs could reflect the genotypes and phenotypes of their parent cells. Following studies confirmed that exosomes could be secreted to serum, urine, cerebrospinal fluid, and all of those indicated that it might be a novel diagnostic maker for cancer [35–39]. It had been confirmed that the miRNA level in TEXs in body fluid showed strong correlation with tumor progression compared to body fluids levels. The related investigations have been reported in glioma, ovarian cancer, lung cancer, hepatocellular carcinoma, gastric cancer, etc [40–44]. Long non-coding RNAs (LncRNAs) also have potential roles to act as new diagnostic and prognostic biomarkers. Recently, researchers have found that lncRNAs expression, which is low in cells, is increased in exosomes derived from glioblastoma, bladder cancer, laryngeal squamous cell carcinoma, colorectal cancer, gastric cancer, liver cancer, etc [38, 45–49]. In a word, detection of the genetic information in exosomes may be a promising way for early diagnosis and surveillance of malignancies.

In recent years, DCs derived exosomes (DEXs) have attracted more attentions due to its immunoregulatory capacity and immunotherapeutic effects in management of cancer. DEX has the ability to present Ags directly and indirectly to T cells, so that they can not only induce T cells dependent antitumor responses but also overcome some technical limitations of immunotherapy based on DCs. The therapeutic potential of DEX mainly depends on its composition. DEX contains abundant MHC Class I and II molecules, costimulatory molecules (CD80 and CD86), the Ig family member ICAM-1, milk fat globule EGF factor 8 (MFG-E8), tetraspanins (CD63, CD81, CD9) and the heat shock protein hsc73 [50–55]. DEX stimulates tumor-specific CTL responses by transferring MHC-peptide complexes to T cells from DCs, which could be facilitated by costimulatory molecule and tetraspanin. ICAM-1, MFG-E8 and hsc73 also can promote immune reaction via inducing T cell-dependent antitumor response [51, 55]. NK cell-dependent antitumor immune response can be activated by IL-15R, TNF, NKp30 ligand BAT3 and NKG2D expressed on the surface of DEX [56–58]. On the basis of those studies, DEX vaccine researches have been carried out for a variety of tumor cells [6, 59–62]. Moreover, phase I clinical trials in melanoma and advanced non-small cell lung cancer showed meaningful results [63, 64]. Application of DEX vaccine may provide a promising approach for cancer prevention and treatments.

### TEXs contain tumor-associated antigen and can uptake by DCs

Exosomes, derived from melanoma, glioma, renal cell carcinoma and other tumor cells, have been proved to contain parental antigens [7–13]. Wolfers et al. reported that tumor cells could secret exosomes, which strongly expressed MHCI, LAMP1, HSP70 and tumor antigens for the first time [7]. Andre et al found that antigens of exosomes could be taken up and cross-presented by MHC-I molecules in HLA-A2+ monocyte-derived DCs [65]. DCs not only express OVA and pMHC-I molecules, but also express both of them after being co-cultured with EXO_EG7_. This was correspond to the previous study that DCs became positive for CFSE or CD45.1 after being incubated with EXO_CFSE_ or EXO_6.1_, and the expression of H-2Kb, pMHC.I, Ia^b^, CD40, CD54 and CD80 in DCs was enhanced after incubation with EXO_OVA_ [66, 67]. While blocking the anti-LFA-1 and anti-DEC205 antibodies or treatment with cytochalasin D could reduce the uptake of exosomes in DCs, suggesting that LFA-1/CD54 and C-type lectin/mannose-rich C-type lectin receptor interactions might be critical for the mechanism of exosomes uptake by DCs [67]. MHC-I (an important factor responsible for capturing antigenic peptide), ICAM-1 (an adhesive molecule which is potentially involved in cell targeting), HSP70 (a crucial chaperones for binding with DCs), and MAGE-1 protein (a glioma specific tumor antigen), were enriched in exosomes from malignant glioma than lysates [8]. Hepa1-6 TEXs express two well-characterized HCC antigens-alpha-fetoprotein (AFP) and glypican 3 (GPC3), and can be taken up by DCs, thereby promoting DC maturation through enhancing the expression levels of CD11c, MHC, co-stimulatory factors (CD80, CD86), and intercellular adhesion molecule (ICAM) [12]. WEHI3B-TEX, characterized by upregulated expression of the crucial receptors CD90, CD44, MFGE8, HSP70 and the tetraspanins CD9 and CD63, can be efficiently taken up by DCs, thus inducing DCs maturation without impairment. TEXs carrying tumor specific antigen can activate maturation of DCs, enhancing anti-tumor immune response.

### DCs loaded with TEXs elicit CD8+ T cell dependent antitumor immunities

In previous studies, it has been proved that TEXs uptake by DCs could induce antigen-specific CTL response. Tumor cells express antigens which can be recognized by CTLs. DCs take up the TEXs containing donor antigens, thereby inducing specific CTL response *in vitro* or *in vivo*. Andre et al. found the DCs loaded with ascitis exosomes from melanoma could increase the number of peripheral blood CD8 positive T cells and stimulate lymphocytes to lyse autologous tumour cells or release interferon-gamma. However, addition of antibodies to MHC class I molecules could inhibit the immune reaction [65]. Ning Bu et al. observed that TEXs uptake by DCs could activate the T lymphocytes to become glioma-specialized CTL, and they demonstrated that CD8 molecules antibodies could inhibit the CTL mediated cytotoxicity to tumor target cell, however antibodies to CD4 could not [8]. In immunotherapy of hepatocellular carcinoma, the number of CD8+ T lymphocytes was increased significantly in serum and tumor tissues of mice treated with TEX-pulsed DCs [12]. Ye Yao et al. found that EXO_EG7_-targeted dendritic cells stronger stimulated CD8+ T-cell to differentiate into CTL effectors *in vivo* [66]. All the researches revealed that DCs loaded with TEXs could increase the numbers of CD8+ T cells, thus enhancing antitumor immunities.

### TEXs show stronger antitumor immunities than tumor derived lysate

In recent years, more and more researches are devoted to explore DC vaccines for cancer prevention and treatments. The tumor lysates represent one of the most frequently used tumor antigens source for DC vaccines. They gain some achievements and also exhibit limited response rate [68]. The relevant studies demonstrated that the effect of TEXs was obviously better than tumor lysates. In Niken M. Mahaweni's study, the median survival for exosome-loaded DC group was longer than tumor lysate-loaded DC group (29.5 with 18.5 days), although mesothelioma-derived exosomes contained less proteins than necrotic tumor lysates [10]. Ning Bu et al. found that the cytotoxicity of exosomes DC-stimulated T cells against glioma cells was significantly greater than the levels in tumor lysate DC-stimulated T cells at all E:T ratios [8]. Hepa1-6 TEXs show superiority to cell lysates in eliciting DC-mediated antitumor immunity *in vitro*, despite of the capability of inhibiting tumor growth and T lymphocytes numbers in tumor tissues *in vivo* and cytolysis rates against hepa1-6 cells *in vitro* [12]. Some studies also showed that significantly stronger tumor suppression was achieved in tumor exosome-loaded DC -treated mice compared with tumor lysate-loaded DC, because of the slower tumor growth, stronger CTL activation and DC taking up rate [7, 69]. DCs loaded with TEXs may be promising for tumor immune therapy without severe side effects and treatment resistance.

### Expansion

In summary, exosomes have huge functions in tumor immunity. Exosomes can be utilized as a strongly efficient antigen compared with traditional tumor lysates because of its strong antigenicity, applicability and conveniences of storing and extracting. TEXs can be separated from blood by specific biomarker, which is suitable for all of the patients including the patients who couldn't tolerate surgical resection of the tumor. DCs capture and process antigens into peptides, and then present them to T cells receptors via major histocompatibility complex (MHC) class I or II thus activate signal 1. The upregulating costimulatory molecules such as CD4+, CD8+ make DCs more mature and have more powerful capability in presenting antigens thus activate signal 2 to release a large number of cytokines, which must be completed by adjuvants. The therapeutic immunity of DC vaccines can be strengthened by pulse of some agents, such as Toll-like receptor (TLR) agonists, CD4+ ligand, CD7+, TNFRSF4 ligand, calcium ionophores, and GITR ligand. For example, recent studies demonstrated that the potent synthetic iNKTs agonist α-galactosylceramide (α-GalCer) combined with DC vaccines could enhance capacity to drive conventional T-cell responses via upregulating immunostimulatory factors such as CD4+ upon encourage iNKT: DC interactions, then express highly effective in immunotherapy of glioma [70]. In addition, the DC vaccine efficacy is variable at present, and the most possible reason may be the immunosupressive response caused by tumor microenvironment. Recent studies show that immune checkpoint inhibitors such as PD-1 antibodies have abilities to relieve immunosuppression and improve the tumor microenvironment [71, 72]. However, application of TEXs loaded DC for cancer therapy has several potential challenges. First, it is well known that malignancy patients only produce dysfunctional DCs at early stages of disease. More attentions should be payed to isolated the eligible DCs with high quality for clinical use. Second, although *in vitro* demonstrated that TEXs could promote DCs maturation, there were a lots of TEXs *in vivo* which might suppress the function of DCs loaded with TEXs. Therefore, the application approaches for DCs use in cancer therapy required further improvements.

In conclusion, the combination of exosomes loaded by DC vaccine with adjuvants and immune checkpoint inhibitors maybe crucial for oncotherapy in the future. Further investigations should be devoted to improve the application strategies of DC vaccine loaded with TEXs for cancer treatments in clinic.
